# Types and Strains: Their Essential Role in Understanding Protein Aggregation in Neurodegenerative Diseases

**DOI:** 10.3389/fnagi.2017.00187

**Published:** 2017-06-16

**Authors:** Wiebke M. Wemheuer, Arne Wrede, Walter J. Schulz-Schaeffer

**Affiliations:** ^1^Institute of Neuropathology, Saarland University Medical CenterHomburg, Germany; ^2^Luxembourg Centre of Systems Biology, University of LuxembourgEsch-sur-Alzette, Luxembourg; ^3^Prion and Dementia Research Unit, Institute of Neuropathology, University Medical Center GoettingenGoettingen, Germany

**Keywords:** α-synuclein, β-amyloid, prion, protein conformation, accelerated seeding

## Abstract

Protein misfolding and aggregation is a key event in diseases like Alzheimer’s disease (AD) or Parkinson’s disease (PD) and is associated with neurodegeneration. Factors that initiate protein misfolding and the role of protein aggregation in the pathophysiology of disease pose major challenges to the neuroscientific community. Interestingly, although the accumulation of the same misfolded protein, e.g., α-synuclein is detectable in all idiopathic PD patients, the disease spectrum covers a variety of different clinical presentations and disease courses. In a more recent attempt this clinical variance is being explained in analogy to prion diseases by different protein aggregate conformations. In prion diseases a relationship between protein aggregate conformation properties and the clinical disease course was shown by relating different prion types to a dementia and an ataxic disease course in Creutzfeldt-Jakob patients. This principle is currently transferred to AD, PD and other neurodegenerative diseases with protein aggregation. However, differences in protein aggregate conformation are frequently addressed as disease strains. The term “strain” also derives from prion research and evolved by adopting the virus terminology at a time when transmissible spongiform encephalopathies (TSEs; later called prion diseases) were assumed to be caused by a virus. The problem is that in virus taxonomy the term “type” refers to properties of the disease agent itself and the term “strain” refers to host associated factors that interact with the disease agent and may moderately modify the clinical disease presentation. Strain factors can be discovered only after transmission and passaging of the agent in a host of a different species. The incorrect use of the terminology confuses disease agent and host factors and hampers the understanding of the pathophysiology of protein aggregate-associated neurodegenerative diseases. In this review article the discoveries are reviewed that explain how the terms “type” and “strain” emerged for unconventional disease agents. This may help to avoid confusion in the terminology of protein aggregation diseases and to reflect correctly the impact of protein aggregate conformation as well as host factor contribution on different clinical variations of AD, PD and other neurodegenerative diseases.

## Introduction

The dysregulation and misfolding of disease-specific proteins and their aggregation are key events during the pathogenesis of neurodegenerative diseases such as Alzheimer’s disease (AD), Parkinson’s disease (PD), Dementia with Lewy bodies (DLB), Creutzfeldt-Jakob disease (CJD) and others. Hence, these processes are under intense investigation as they may serve as potential therapeutic targets. Common mechanisms seem to underlie these neurodegenerative diseases: (1) they all are associated with the aggregation of physiologically occurring proteins like hyperphosphorylated tau, β-amyloid-cleavage fragments of the amyloid-precursor protein, α-synuclein, prion protein and some other proteins; (2) aggregates accumulate in central nervous system (CNS) tissues; (3) over time, a spread of aggregates is detectable, involving more and more structures of the CNS; and (4) the clinical symptoms of patients reflect the dysfunction of brain regions in which the aggregation process is detectable.

All these neurodegenerative diseases are associated with the corruption of a physiologically occurring protein into a pathological conformation that is prone to form aggregates. This principle was first discovered in prion diseases or transmissible spongiform encephalopathies (TSEs). They occur in both humans and a number of animals and are always transmissible by the intracerebral inoculation within the same species and in principle between species. The pathological prion protein was the first transmissible agent that was suspected to be proteinaceous and have the ability to self-propagate in the CNS. Therefore, when the concept of protein aggregate propagation was applied to the aggregating proteins in PD, AD, DLB and other diseases that were considered to be non-transmissible, a “prion-like” spread of aggregates was frequently postulated (Acquatella-Tran Van Ba et al., 2013; Walker and Jucker, 2015; Hasegawa et al., 2017). The mechanism of spread is also addressed as “accelerated seeding” (Beekes et al., 2014).

The aggregation of proteins with an identical primary amino acid sequence can lead to different clinical disease courses. In prion diseases, this is explained by different secondary and tertiary conformations of the misfolded protein. In sporadic CJD, two prion types that differ in their conformation after proteinase K digestion are indeed associated with distinct clinical, pathohistological and biochemical features (Parchi et al., 1999, 2000; Wemheuer et al., 2009). It is proposed that conformational differences are responsible for the clinical variety in other neurodegenerative diseases in a very similar manner as in prion diseases. Unfortunately, the proposed conformational differences that are associated with the accumulation of tau, β-amyloid or α-synuclein have been frequently addressed as “strains” (Watts et al., 2014; Melki, 2015; Taniguchi-Watanabe et al., 2016), a term that also derives from the prion field. It originally referred to stable properties in incubation time and brain lesion pattern that could be observed after passaging scrapie isolates in a different species, usually rodents (Fraser and Dickinson, 1968). The term “strain” is simply an expression of the taxonomy employed by virology, because at that time TSEs were thought to be slow virus diseases (Sigurdsson, 1954). Importantly, a strain unifies the properties of the transmissible agent with those of the host, which is why different mouse lines produced different scrapie strains. Similar experiments that investigate the properties of tau, β-amyloid and α-synuclein aggregates from different clinical sources or pre-formed aggregates in animal models and cell culture have been successfully conducted (Luk et al., 2012; Bousset et al., 2013; Clavaguera et al., 2013; Masuda-Suzukake et al., 2013; Aulić et al., 2014; Sacino et al., 2014; Sanders et al., 2014; Watts et al., 2014; Iba et al., 2015; Peelaerts et al., 2015). In some animal models, the passaging of transmitted α-synuclein, tau or β-amyloid in the new host proves indeed the existence of strains (Sanders et al., 2014; Watts et al., 2014; Prusiner et al., 2015). The characterization of tau, β-amyloid or α-synuclein isolates from humans, however, should describe types, not strains. An incorrect use of terminology leads to wrong conclusions i.e., regarding cause and consequence in the interaction of protein aggregates in pathophysiological processes of the cell. The current review article aims to explain, how the terms “type” and “strain” evolved in diseases with an unconventional agent and traces the changes in the perception of these diseases within the last decades.

## The History of Prion Strains

### How it Started: The Transmissibility of Scrapie Suggests that the Infectious Agent Is a Virus

After Cuillé and Chelle (1938) had succeeded in 1936 in transmitting scrapie from one sheep to another by the intraocular inoculation of spinal cord material (Cuillé and Chelle, 1936), they concluded in 1938 that the infectious agent must be a virus. They reproduced their transmission experiment and showed that material passed through an antibacterial filter was still able to cause the disease (Cuillé and Chelle, 1938). The fact that, during later experiments in the 1960s and 1970s, the scrapie agent was able to suddenly change its properties upon serial transmission in different rodents, i.e., neuropathological lesion profile and incubation period (see below), was in part interpreted as a genomic mutation of the scrapie agent. This further supported the notion that the infectious agent was either a virus or at least had an independent genome (Bruce and Dickinson, 1987). In due course, the assumption that scrapie was a viral disease became responsible for the emergence of the use “scrapie strain”, as analogous to the term “virus strain”.

### Characterization of Scrapie in Analogy to Viruses

In virology it is common knowledge that virus strains can be characterized by comparing their symptomatology in differential hosts (Shukla and Ward, 1989). Here the strains are an expression of the variants in a virus species or type (Matthews, 1985). The new host may influence the pathogen, and changes in the pathogen are known as host passage effects (Yarwood, 1979); thus the comparison of different isolates in the same host is of pivotal importance. The result of a virus infection is determined by the genome of the virus, the genome of the host and the relationship between the two (Dijkstra, 1992). This was common knowledge in the field of virology when the passaging of sheep scrapie in certain inbred mouse lines succeeded and led to distinct disease variants (see below). Therefore, these variants were referred to as scrapie strains. Different scrapie strains showed specific reproducible interactions with defined inbred mouse lines, i.e., unique incubation times and histopathologic profiles.

Today, the genome provides the basis in modern virology for following the path of evolution and provides evidence for how closely isolates are related, aiding the classification into virus species/types and strains (Gibbs, 2013). The classification is an ongoing task, which is pursued by specialist groups in the different fields of virology and the International Committee on Taxonomy of Viruses (ICTV)^1^; and while the importance of distinguishing subspecies, strains and isolates is recognized, these lower levels are not formally classified by the ICTV (Murphy et al., 1999; Büchen-Osmond, 2003).

In prion diseases there is no genome to help the classification. All attempts to isolate and sequence a nucleic acid from the infectious agent have been in vain so far (Meyer et al., 1991; Riesner, 1991), even though certain DNA or RNA fragments seem to take part in the misfolding of the physiological prion protein into its pathological isoform (Deleault et al., 2010). If the infectious agent contains a nucleic acid, it seems to be extremely well protected by the proteinaceous part of the prion, and the strain-specific properties of the agent may well be determined exclusively or additionally by the properties of the misfolded protein. As a classification with the help of homologous sequencing is therefore impossible, a clear definition of prion strains and prion types will need different criteria. A close look at how the terms evolved and what possibilities exist today to examine prion diseases in original and new hosts might help with this task.

### The First Scrapie Strains Were Observed after Transmitting the Disease to Goats

**Figure 1:**
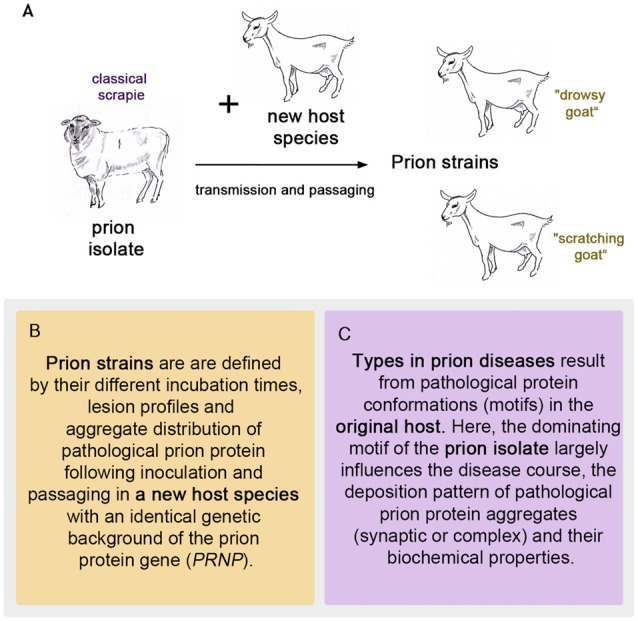
The first prion strains were isolated in goats after passaging sheep scrapie in them, as pictured in **(A)**. Definition of strains **(B)** and types **(C)** in prion diseases.

To follow the evolution of the term **scrapie strain** we go back to the time that followed the pioneering achievement in scrapie research by Cuillé and Chelle (1936). At the Moredun Institute in Edinburgh, scrapie research was pursued extensively in the original host, the sheep, mainly by D. R. Wilson. His former colleague I. H. Pattison recalls in his personal view of scrapie in 1971: “*Wilson accepted the conclusion reached by Cuillé and Chelle that it was a virus disease, and he set about looking for the virus. (…). Wilson’s achievements were remarkable when one remembers that his only method of detecting the transmissible agent was by inoculating sheep only about 25% of which were susceptible to the disease after up to a year’s incubation period”* (Pattison, 1971). Therefore, an animal model with a better attack rate and shorter incubation periods was highly desirable to study this new disease. It was I. H. Pattison who found a new animal model when he was engaged in scrapie experiments at the Compton Institute—the second stronghold of scrapie research in the United Kingdom at that time (Field, 1976). Goats, intracerebrally inoculated with sheep scrapie showed a susceptibility of 100% (Pattison et al., 1959). Even though the general susceptibility of goats to scrapie was not entirely new (Cuillé and Chelle had succeeded in transmitting sheep scrapie to two goats by intraocular inoculation Cuillé and Chelle, 1939), this was indeed very good news when compared to sheep scrapie. The goat assay offered, for example, the possibility of evaluating the decrease in infectivity after different treatments of scrapie inoculates (Pattison and Millson, 1961). Importantly, the infected goats displayed different clinical features. Dominating were two clinical syndromes referred to as “nervous” (later “drowsy”) and “scratching”, which could be related to different inocula (Pattison and Millson, 1961). Usually one of the clinical syndromes occurred in the infected goats. Notably, the scratching syndrome had emerged only after five intracerebral passages of scrapie in goats. Pattison and Millson conclude these observations with *“It is suggested that certain ‘strains’ of the scrapie agent will produce the nervous syndrome, while others will produce the scratching syndrome”* (Pattison and Millson, 1961). Thus the first **scrapie strains** were observed and named after passaging sheep scrapie in goats (see Figure 1A).

### The Transmission of Scrapie to Mice Becomes Successful

The difference in these strains became even clearer when Chandler (1961) infected three different laboratory mouse lines with “drowsy” and “scratching” goat scrapie material, respectively. The Swiss white mice infected with “drowsy” goat material showed neurological symptoms and pathological lesions in the brain after 7.5 months. Later the C57BL and C.B.A. mice infected with “drowsy” goat material developed the disease as well (Chandler, 1962). At this time point, “scratching” scrapie goat material did not seem to transmit to mice. It was a huge success however, that scrapie had been transmitted to mice at all, and with this a new era in scrapie research started, as mice were easier to maintain than goats and had shorter incubation periods. Chandler’s initial mouse experiments at the Compton Institute were repeated and continued. Passaging the scrapie agent twice in Swiss white mice resulted in shortened incubation times of 3–4 months. Chandler concluded, “*Thus there was evidence of adaption of the agent in mice”* (Chandler, 1962). The transmission of the “scratching goat” scrapie strain to mice (Zlotnik and Rennie, 1963), and also the direct transmission from sheep scrapie to mice (Zlotnik and Rennie, 1962), turned out to be successful as well in the course of the adaption process. While clinical signs were absent or took a long time to appear during the first passage (although typical scrapie lesions in the brains were present), the second passage remarkably shortened incubation periods, resulting in fatal clinical disease for the mouse passaged “scratching goat” and some sheep scrapie isolates. Evidently, there was a **species barrier** to overcome. More rodent models were developed. Scrapie was transmitted to rats (Chandler and Fisher, 1963) and golden hamsters (Zlotnik, 1963), chinese hamsters, voles, gerbils and guinea pigs (Chandler and Turfrey, 1972). Different routes of infection were tested (Zlotnik and Rennie, 1962) and the mouse material was transmitted back to goats and sheep to verify that this mouse encephalopathy indeed resembled scrapie (Zlotnik and Rennie, 1963, 1965). The investigation of the behavior of the scrapie agent in different species became a main focus of scrapie research.

### Scrapie Strain Characteristics in Inbred Mice Depend on Scrapie Source and Mouse Genome

For the assessment of mouse brains A. G. Dickinson, H. Fraser and their colleagues at Edinburgh developed “*a system of scoring the brain pathology which depended on an assessment of the intensity of neuronal vacuolation and status spongiosus in paraffin sections stained with haematoxylin and eosin*” (Fraser and Dickinson, 1968). They applied this method to narrowly defined brain structures and focused on the development of lesions in different mouse lines after the inoculation with the same scrapie source. While the outcome differed considerably between the mouse lines, it was highly reproducible within one line. They concluded, “*With a single agent and identical challenge, the distribution of lesions during and at the end of the incubation period is under the influence of the host genotype*.” (Fraser and Dickinson, 1968). A genetically well-defined host can thus be used to detect differences between scrapie agents. Additional important parameters to characterize a scrapie agent in a new host were the incubation period itself (which stabilizes after serial passaging), clinical syndrome and agent concentration. While in a specific mouse line the incubation period depends on the scrapie strain and the initial infecting dose, the characteristic lesion profile is solely determined by the scrapie strain (Bruce, 1993). Two especially clear scrapie strains in mice at that time were 22A and ME7 (Dickinson and Meikle, 1971).

### Influence of Recipient’s Genome on Strain Characteristics

By crossing and backcrossing two inbred mouse lines and inoculating the mice with ME7 (Dickinson et al., 1972), identified a gene in mice that determined the incubation period, which they called *sinc* for *s*crapie *inc*ubation. *“The two alleles which show no dominance are designated s7 for the one which shortens the incubation period and p7 for the one which prolongs it”* (Fraser and Dickinson, 1968). Heterozygote mice had intermediate ME7 incubations times. The 22A scrapie strain behaved in the opposite way in the same homozygous mouse strains and produced not intermediate but the longest incubation periods in heterozygote mice. The incubation periods of s7s7, p7p7 and s7p7 mice became a useful tool to discriminate a variety of scrapie strains in mice in addition to the lesion profile (Dickinson and Meikle, 1971; Bruce et al., 1991; Carp and Callahan, 1991). Nowadays, it is known that the *sinc* gene alleles s7 and p7 correspond to two polymorphisms in the murine prion protein gene: L108/T189 for s7 and F108/V189 for p7 (Westaway et al., 1987). Regardless of the latter though, it was this “old” established method of characterizing scrapie strains in inbred mice that helped to identify bovine spongiform encephalopathy (BSE) as the likely origin of human variant CJD (vCJD). BSE was first described in Great Britain in the 1980s. BSE and vCJD isolates caused more or less identical lesion profiles and incubation periods upon passaging in the same inbred mouse strains (RIII, C57BL and VM) and one cross (C57BL × VM; Bruce et al., 1997). This “BSE signature” (Bruce et al., 1994) was also found upon transmission of TSEs in domestic cats (Fraser et al., 1994) and two exotic species of ungulates (Kirkwood and Cunningham, 1994), indicating that the BSE agent had crossed the species barrier easily more than once. The example of the “BSE signature” demonstrates that a common TSE strain pattern in mice after transmitting different animal TSEs certainly supports the conclusion that the same source caused the original TSEs in their hosts. Since the host genotype determines the properties of the prion strain in combination with the properties the isolate brings with it from the original host, the original TSEs and the resulting strains in mice should be viewed as two separate diseases. Regarding incubation period and neuropathological characteristics in a particular murine PrP genotype for passaging TSEs, M. E. Bruce summarizes, “*A TSE strain is defined from this set of stable properties, rather than its origin”* (Bruce, 2003), and Aguzzi et al. (2007) puts the strain definition in a nutshell by stating, *“distinct prion strains can only be identified by bioassays that detect the transmission of strain characteristics in new hosts”*.

### Hypothesis of an Independent Genome of the Scrapie Agent

In the 1980s, Bruce and Dickinson (1987) strongly supported the idea of an independent genome of the scrapie agent with their research. In certain well-defined scrapie strains a “breakdown” could be observed if very high doses were used to passage the agent in mice. This meant that the scrapie agent changed its properties (incubation period and lesion pattern) dramatically, which they and others thought was caused by a mutation of the agent and subsequent selection by the host. *“This phenomenon occurred repeatedly using six independent isolates of scrapie (31A, 51C, 87A, 125A, 138A and 153A), but was never seen with at least 15 other scrapie strains which were being used in a wide range of experiments in the same laboratory.”* (Bruce and Dickinson, 1987). As an example, scrapie strain 87A gave way to strain 7D. Importantly, 87A and other strains had been cloned before challenging the “breakdown”, meaning that by several sequential passages using the minimum-infecting dose possibly present, minor strains had been removed (Bruce and Fraser, 1991). This was meant to ensure that the emergence of new strains was caused by a mutation followed by selection and not by a simple selection process. Mutations were assumed to occur on a genomic level and provided therefore evidence of an independent genome (Bruce and Dickinson, 1987).

### Strain Selection by the New Host

Apart from the “breakdown” of scrapie strains, selection also seemed to play a role in the species barrier. It has already been mentioned that upon first transmission of a scrapie agent into a new species, an adaption of the agent takes place, meaning a longer incubation period, which will shorten and stabilize upon serial passaging in the new host. If the adaption takes longer than two passages in the new host species, most likely a strain selection takes place, as Kimberlin and Walker (1978) could show with their experiments. Similar conclusions were reached by Carp and Callahan (1991) by transmitting five sheep scrapie isolates to s7s7 and p7p7 mice, and scientists from the Neuropathogenesis Unit in Edinburgh with their extensive experience of mouse scrapie strains (Bruce, 1993). This implies that an isolate usually contains a mixture of strains or agent variants. One strain will be preferred by the host and therefore dominate the neuropathological outcome by presenting its characteristic lesion profile upon passaging in the new host.

### The Multimeric Replication Site Model

The idea that strains are competing in one host led to a theory that became known as the “scrapie replication hypothesis”, according to which “*The great diversity of scrapie incubation periods and modes of gene action of sinc can be explained in terms of the multimeric replication site model”* (Dickinson and Fraser, 1977). In their experiments Dickinson et al. (1972) observed prolonged incubation periods in mice that were first inoculated with scrapie strain 22C and after several weeks with strain 22A. They put this effect down to strain competition, which only occurred once the slower replicating strain 22C had presumably “*blocked replication sites”* for the faster incubating strain 22A. In a similar manner they were able to prolong ME7 incubation times with 22A, but a longer interval (180 days) between administrations of the two inocula was necessary. It was due to this long interval that a conventional immunological response was considered unlikely to offer any plausible explanation for the prolonged incubation periods and the theory of strain competition was favored (Dickinson et al., 1972). Yet, how exactly the replication of the infectious agent works remained (and still remains) unknown.

### Hypothesis of Conformational Variants of an Infectious Protein

The concept that each isolate consists of a mixture of strains has been very much supported by the recent research of Weissmann (2012) and his group, who successfully work with prion propagation in mouse cell lines. Their cell panel assay works on the basis of the differential susceptibility of cell lines to various prion strains in addition to the susceptibility of some strains to drugs (Li et al., 2010). The cell panel assay can perform serial passaging much faster than animal models (Weissmann, 2012). Like most in the field, Weissmann (2012) and his colleagues accept that the infectious agent of TSEs is made up mainly, if not entirely, by protein as Prusiner (1982) suggested when he coined the term “**pro**teinacious **in**fectious particle” (abbreviated “prion”) for this kind of agent. With this approach, the phenomena of mutation and selection observed with the cell panel assay can be explained as follows: “*prion populations (…) are composed of a variety of conformational variants, each present at a low level; when the environment changes, the most efficiently replicating variant becomes the predominant component of the population, which then constitutes a distinct sub-strain”* (Weissmann, 2012). The underlying understanding with this explanation is the idea that prion strains differ in their protein conformation, as shown, for example, by Safar et al. (1998) for the pathological/scrapie prion protein (PrP^Sc^) of eight hamster-adapted prion strains with a conformation-dependent immunoassay. Two of the distinct hamster prion strains, called “Hyper” and “Drowsy”, had been isolated in golden hamsters after infection with transmissible mink encephalopathy (TME) of the same source, the Stetsonville isolate (Bessen and Marsh, 1992b), providing yet another example of host selection (very similar to the occurrence of the two scrapie strains in goats after serial passaging). The conclusion Safar et al. (1998) drew from their conformation-dependent immunoassay experiments was that “*biological properties of prion strains are ‘enciphered’ in the conformation of PrP*^Sc^”.

### Selection of Conformational Variants from the “Prion Cloud” after Transmission to a New Host

Consistent with this concept, the adaption to the new host is a process which “*implies as a first step accretion of PrP^c^* (= physiological/cellular prion protein)* from the recipient host to the incoming PrPSc seed, which may be a very inefficient process if the amino acid sequence of the host PrP entrains a spectrum of conformations that are poorly compatible with that of the seed.”* (Weissmann, 2012). Therefore, if the host does not favor the initially most frequent conformational variant in the mixture, another might come to dominate the population after transmission. The pressure of selection towards drug resistance in the cell panel assay seems to promote this tendency (Li et al., 2010), but *in vivo* the transmission to a new host is likely to serve the same purpose. At this point, the new host influences a strain on the level of the primary amino acid sequence and by selection, “picks” a suitable conformer from the prion cloud (Collinge, 2010; Li et al., 2010). Other host-specific factors may have a role in the outcome of the strain in the new host as well. For the presence of these conformational variants, Li et al. (2010) offer two explanations: either conformational variants exist in the population at a low level before exposure to the drug (swainsonine) or they are generated during exposure to the drug. Swainsonine (“swa”) is a drug that does not reduce cell growth, but by inhibiting Golgi α-mannosidase II leads to misglycosylations that reduce the accumulation of “swa-sensitive”, but not “swa-resistant” prions (Li et al., 2010). For the above-described breakdown of certain scrapie strains in mice this would mean that the “mutation” occurs due to either existing and then favored conformers in the (cloned) sample or the same new and presumably more stable conformers arise each time during the propagation process under reproducible conditions and are followed by selection. It is conceivable that during the misfolding process, also aberrant conformers of pathological prion protein may be produced that differ from the conformational variant that is mainly propagating and forming the fibrils. How one of those could end up dominating the strain properties in a new host is explained by the idea of deformed templating, which postulates that *“…a state with one cross-beta-folding pattern can seed an alternative self-replicating state with a different folding pattern. Such seeding is possible if the parent and daughter states share common structural motifs that link the hybrid structure”* (Makarava and Baskakov, 2013), and “*while the majority of the newly generated variants might not be effective in replicating, a variant that fits well to the new environment will eventually emerge through multiple trial-and-error seeding events”* (Makarava and Baskakov, 2013). So an aberrant conformational variant that arose during misfolding can under certain conditions seed, propagate and become part of the prion cloud. From the cloud it could become the dominating conformer through selection, thus forming a new prion strain with its own distinct properties. Analogous to point mutations in viruses, this could indeed be seen as a “prion mutation”.

### The Replication Site Model Revisited in the Light of Conformation

Strain competition can be observed in the hamster model with the two TME-derived strains Hyper and Drowsy in a manner similar to that described in the 1970s with slow and fast replicating mouse scrapie strains (see above). Shikiya et al. (2010) worked with co-infection experiments in the hamsters *in vivo* and the protein misfolding cyclic amplification (PMCA) *in vitro*. They showed that the slower replicating strain Drowsy does not overrule Hyper by simply converting all of the available physiological prion protein (PrPC) into its pathological isoform (PrPSc). They hypothesize, *“that Drowsy PrPSc binds to and sequesters PrPC, and/or other cofactors required for conversion, rendering it unavailable to Hyper PrPSc for conversion.”* (Shikiya et al., 2010). Such co-factors could be e.g., RNA molecules or glycosaminoglycans and *“these factors, in combination with PrPC, may form the functional replication site”* (Shikiya et al., 2010). The replication site model might play a role in strain selection, with the host genetics determining factors for the outcome of the respective strain in more ways than we currently understand.

### The Confusion in Terminology Occurred with the Discovery of Polymorphisms in Sheep *PRNP* and Atypical Scrapie/Nor98

Scrapie in sheep seems to be a rather inhomogeneous prion disease on a molecular level, given how many scrapie strains in mice could be isolated in contrast to e.g., classic BSE, which always resulted in the same two prion strains in mice: 301A and 301V (Bruce et al., 2002). The discovery of different prion genotypes in sheep (Belt et al., 1995; Hunter et al., 1997) helped, on the one hand, to explain the varying susceptibility of individual sheep and sheep families to scrapie, which had puzzled Wilson and his colleagues in the early transmission experiments of scrapie research (see above); on the other hand, it misleadingly introduced the term “prion strain” in connection with the original host species—the sheep. Even the neuropathologist W. J. Hadlow, who had made the connection between human “Kuru” and sheep scrapie in 1959, commented in 1999 “*The meaning of these strains in the natural occurrence of scrapie in sheep and goats is still unclear, though I’m sure they partly explain the varied phenotypic expression of the disease, at least in sheep, not only clinically but also neuropathologically. Given the manipulation most strains have undergone, some may be more laboratory-derived or laboratory-generated artifacts than representatives of extant strains in the wild, in the real world.*” (Hadlow, 1999). Terms like “scrapie strain variation in the natural host” (Bruce, 1993) and “scrapie field strains” (Moore et al., 2008) did not help to keep the term “scrapie strain” separate from the original host species, either. So when a new type of sheep scrapie was found in 1998 in Norway by Benestad et al. (2003); it was indiscriminately called a new scrapie strain, although it had not yet been compared to other scrapie strains in inbred mice—the essential requirement for being able to define a strain. Unfortunately, Nor98/atypical scrapie does not transmit to the inbred wild type mouse lines (Bruce et al., 2006) that have proven so useful in establishing e.g., the BSE signature and in characterizing many classic scrapie strains. Other laboratory techniques, such as the glycoform analysis of proteinase K (pK) digested prion in Western blotting, added to the confusion in terminology, because they were applied to distinguish different forms of TSEs in their original host as well as different scrapie strains in their new host. Somerville et al. (1997) pointed out that while the examined mouse strains each show a unique glycosylation pattern for themselves, the prion source does not necessarily influence it. This was the case for BSE and vCJD, which share the same strong diglycosylated prion protein fraction (Collinge et al., 1996). While this also helped to identify BSE as the likely origin of vCJD (Hill et al., 1997) in addition to the lesion profile in inbred mouse strains (Bruce et al., 1997), Somerville et al. (1997) showed that this was not a criterion to be relied on.

### Summary: What Defines a Strain

**Figure 2:**
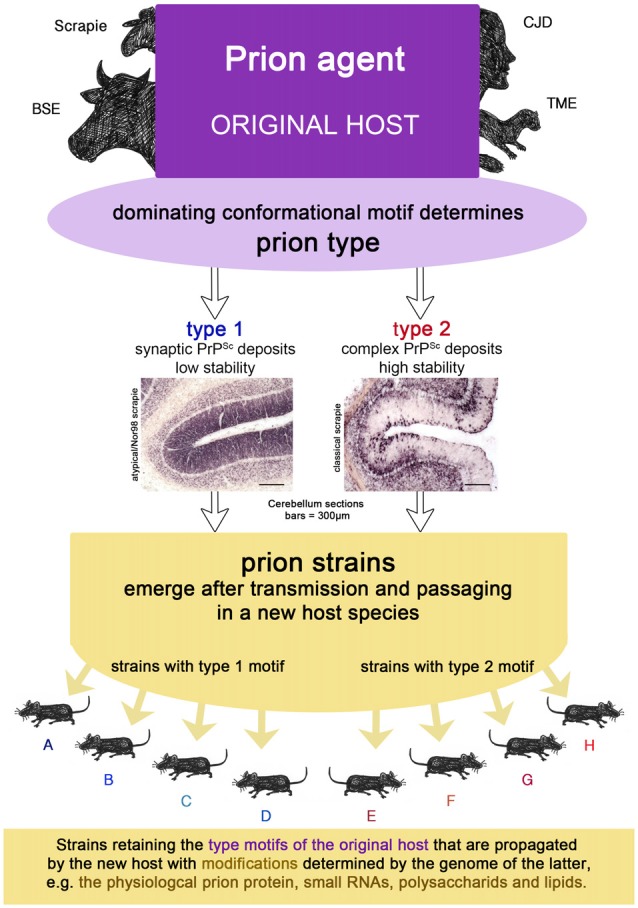
The prion isolate from the original host can be transmitted to a new host species in which prion strains can emerge upon passaging. Strains retain the type motifs of the original host that are propagated by the new host, but they also undergo smaller modifications due to the genetics of the new host.

Prion strains emerge upon transmission and serial passaging of a prion isolate in a species that differs from the one the prion isolate came from (see Figures 1B, 2). The properties of the strain are therefore a product shaped by interaction between the new host and the information enciphered in the conformation of the original prion isolate. The host influence relies on the amino acid sequence of the cellular prion protein as the substrate and most likely other factors, e.g., RNAs, glycosaminoglycans, chaperones and lipids (Baron and Caughey, 2003; Geoghegan et al., 2007). Moreover, if the new host environment does not favor the dominating conformational variant of the original isolate, a different one (present or emerged through deformed templating) will eventually dominate the strain.

## The Difference Between Types and Strains

### Conformational Motifs of the Pathological Prion Protein in the Original Host Determine Prion Types

As mentioned above, in 1982 Prusiner (1982) called the scrapie agent *proteinaceous infectious particle* and the abbreviated term *prion* has been used widely ever since. But the idea that the scrapie agent must be devoid of a nucleic acid that is worthy of a virus was developed a lot earlier than that. Alper et al.’s (1967) experiments, in which she irradiated the scrapie agent with wave lengths that would inactivate a viral nucleic acid, convinced many contemporary researchers in the field that the scrapie agent was able to replicate without a nucleic acid (Pattison, 1971). This heretical idea contradicted the central dogma of molecular biology by Crick (1970), who placed the “protein → protein transfer” among the improbable ones. How a protein could propagate itself, was elucidated by Griffith (1967) in a publication following T. Alper’s challenging article in 1967. Griffith points out that “*.…there is no reason to fear that the existence of a protein agent would cause the whole theoretical structure of molecular biology to come tumbling down”* (Griffith, 1967). His “second way” comes very close to how prion propagation is considered to work these days by many researchers in the field: if condensation nuclei (a seed for infection) are provided, the misfolding of an already present protein into a conformational isoform can take place in the course of polymerization. Gibbons and Hunter (1967) suggested that “*the appearance of the agent of scrapie in a cell may represent an alteration in the basic three-dimensional configuration of a commonly occurring unit membrane structure”*. In their scenario it did not necessarily have to be a protein, but could also be a polysaccharide structure. Alper et al. (1978) continued her experiments and by exposing the scrapie agent to ionizing radiations in the presence or absence of oxygen, she and her colleagues came to the conclusion that membranous systems were likely to be part of the agent. The intriguing idea of a replicating proteinaceous pathogen had gained a foothold and in 1982 Bolton et al. (1982) reported a cellular protein that “purified with the scrapie prion”. But how could information, e.g., of different strains in inbred mice sharing the same genotype, be encoded in a protein, if a nucleic acid was not responsible for this? Charles Weissmann explained this phenomenon by a selection of different conformational variants obtained through serial passaging. However, the notion that different conformations of the pathological prion protein provide distinct clinical properties was initially put forward by Bessen and Marsh (1992b) who had observed, as stated above, the emergence of two distinct prion strains after passaging the TME Stetsonville isolate in golden hamsters. They differed regarding lesion patterns and clinical symptoms, as well as the relative solubility, protease sensitivity and migration pattern of the protein after digestion with pK. The differences in size observable by Western blotting allowed the conclusion that different conformations had led to different cleavage sites (Bessen and Marsh, 1992a). In human prion diseases, the observation that different disease phenotypes can be correlated with differences in size of the pK-cleaved pathological prion protein (and therefore likely different conformations) was made first by Monari et al. (1994) for FFI and the genetic form of CJD, which shares the same mutation (D178N). Parchi et al. (1996) showed soon after, that in a similar fashion, two forms of prion protein can exist in one human disease, i.e., sporadic CJD. Since these disease forms were not discovered as new strains upon passaging in a species different from the original one, but directly in humans, Parchi designated these two forms **prion types**.

### Prion Types in Sporadic Creutzfeldt-Jakob Disease

Sporadic CJD type 1 comprises an unglycosylated fragment of 21 kDa, while in CJD type 2 it has a size of 19 kDa after pK digestion (PrPCJD type 1 or 2). The latter also holds true for pK-digested variant CJD and FFI prion aggregates, which are easily distinguishable from PrPCJD type 2 by their characteristic glycosylation profiles. Experimental inoculation of sporadic CJD type 1 and FFI homogenates into chimeric murine-human prion mice revealed that the 2 kDa difference in pK cleavage size is retained and therefore it must be a feature of the original isolate and not the new host (Telling et al., 1996). In sporadic CJD the prion type in combination with the methionine/valine (M/V) polymorphism at Codon 129 of the cellular prion protein was related to six disease phenotypes in a large cohort of 300 CJD cases (Parchi et al., 1999). However, the PrPCJD type proved to be the main determinant for whether the deposition pattern of pathological prion aggregates was synaptic (Kitamoto and Tateishi, 1994) or complex, i.e., comprising cell-associated, perivacuolar, perineuronal and/or plaque-like prion deposits. Distribution of protein aggregates as well as the presence of amyloid plaques in the brains of CJD patients with PrPCJD type 2 depended also on the prion genotype. At this point, a type in human sporadic CJD was determined by size of the pK-resistant fragment PrPCJD in western blot analysis, the protein aggregate deposition pattern and the dominating clinical symptoms.

### Types with Comparable Properties in Prion Diseases Exist Across Species

Subsequent experiments conducted to determine the stability of pathological prion aggregates against GdnHCl identified additional type-related differences (Wemheuer et al., 2009). Regardless of the genotype, the stability of sporadic CJD type 1 aggregates in these experiments did not exceed the unfolding capability of 2M GdnHCl, while prion aggregates from all sporadic CJD type 2 cases were still detectable at 3M or even higher concentrations of GdnHCl. Interestingly, there were striking parallels between the two main forms of sheep scrapie and human CJD with regard to the prion deposition pattern and stability against GdnHCl. Atypical/Nor98 scrapie shared with CJD type 1 the synaptic deposition pattern and a lower stability against unfolding by GdnHCL than CJD type 2 and classic scrapie. Additionally, classic scrapie and CJD type 2 have a complex deposition of prion aggregates in common. It is well known that variant CJD, with its pK-digested unglycosylated fragment of 19 kDa (Parchi et al., 2000) and complex deposition pattern featuring the characteristic florid plaques (Bruce et al., 1997), fits the type 2 scheme, even though it differs from the sporadic type 2 CJD form in its prion deposition pattern. In addition to the first-described form of BSE (in the following referred to as classic BSE), two further forms have been detected, which occasionally occur in older individuals and are considered to resemble sporadic prion diseases in cattle: bovine “amyloidogenic” spongiform encephalopathy, BASE or L-type BSE (Casalone et al., 2004), and H-type BSE (Biacabe et al., 2004). Classic BSE, BASE and CWD in deer also resemble a type 2 disease with regard to their deposition pattern, which comprises cell-associated, perivacuolar, perineuronal and/or plaque-like prion deposits. Zanusso (2010) showed with 2D gel electrophoresis that variant CJD, in particular, is similar to classic BSE, H-type BSE to sporadic CJD type 1 and BASE to a subform of sporadic CJD type 2 forming plaques, i.e., CJD type 2 in individuals heterozygous for the M/V polymorphism at Codon 129, also referred to as MV2. These findings indicate that the sporadic forms in both humans and cattle have a similar PrP signature, while classic BSE and variant CJD are also similar to each other, as expected, since variant CJD was derived from BSE transmission to humans. All this argues in favor of the explanation that a significant difference in conformation seems to provide a conformational motif that is associated with important clinical, biochemical and neuropathological features of the respective infectious agent.

### Identifying Conformational Motifs that Characterize Types

For CJD type 2, such a structural motif has been almost certainly identified by Zwecksstetter’s group using human prion stop mutants. It consists of an amyloid core involving amino acids 109–142, in which the side chain of the polymorphic residue 129 is deeply buried (Skora et al., 2013). While the replacement of Met129 by Val129 enhances the prion aggregation, a replacement with other amino acids results in no seeding effect. The difference in conformation that determines the pK cleavage site in sporadic CJD is situated a little more towards the N-terminus, i.e., type 1 is cleaved around the 82th and type 2 around the 97th amino acid (Parchi et al., 2000). Using filtration methods, Kobayashi et al. (2005) have shown that CJD type 1 aggregates are much smaller than those of CJD type 2, which fits the observation made concerning the stability of aggregates towards GdnHCl and the neuropathological deposition pattern of pathological prion protein. Whether it is one motif that determines all the characteristics of type 2 in human prion disease (aggregate size, deposition pattern, stability and pK cleavage site) or whether it is more than one, remains to be elucidated.

One or more conformational motifs that lead to an obvious difference in the deposition pattern of prion aggregates (synaptic or complex), the aggregate size and their stability towards denaturation are suitable tools to characterize a prion type in a prion disease of the original host. Different pK cleavage sites, though helpful in one species to distinguish types like classic and atypical/Nor98 scrapie from one another, add to the complexity of interspecies comparisons of prion disease.

### The Transmission Route Might Cause Type Selection

**Figure 3:**
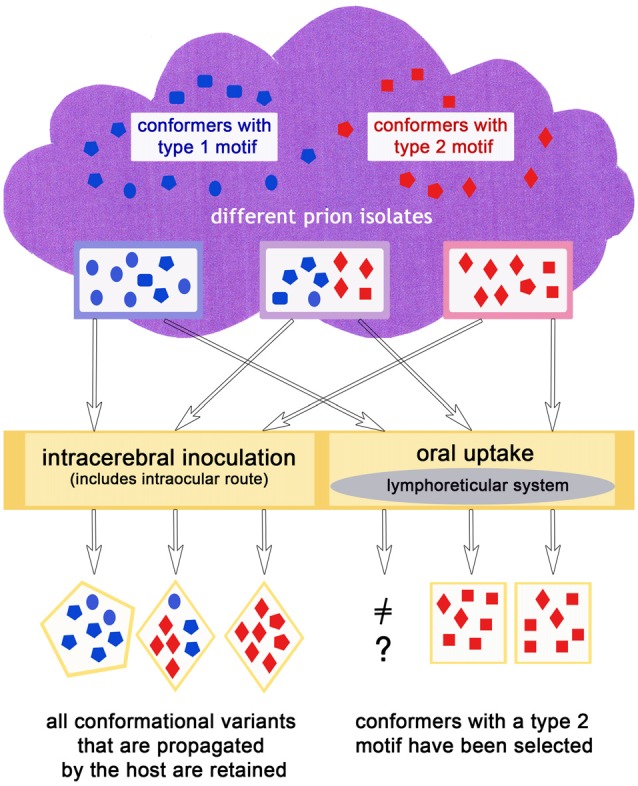
The infection route could influence the outcome of the dominating conformers in the same or a different host species, if for example, an oral uptake leads to a preferred propagation of type 2 conformers.

It does not seem to be a coincidence that prion diseases that have spread in populations like classic scrapie, CWD, BSE and variant CJD show histopathological features relating to a type 2 prion disease, in agreement with the complex deposition patterns observed in CJD type 2 by Parchi et al. (1999). In a scenario in which primarily type 1, but also type 2, conformers appear in sporadic diseases (which could mirror the situation in sporadic CJD), or rather both exist to a certain degree according to the cloud model (even in diseases classified as type 1 such as atypical/Nor98 scrapie) an explanation for this lies at hand: a conformer selection in favor of type 2 conformers takes place upon natural transmission (i.e., most likely oral) to mammals of the same or a different species and in doing so, the disease acquires the ability to spread in a population more easily. As matter of fact, sporadic prion diseases can comprise more than one prion type. For example in sporadic CJD, mixed cases with prion type 1 and 2 occur quite frequently (Parchi et al., 2009). That a “type shift” is possible in principle is supported by recent results in which a sheep, inoculated with atypical/Nor98 scrapie even via the intracerebral route, developed a form of classic scrapie (Simmons et al., 2015). Also, bovine transgenic mice injected with H-type BSE could either develop the mouse form of H-type (a type 1 disease) or classic BSE (a type 2 disease; Torres et al., 2011). A selection of conformers, as explained previously, provides the most plausible explanation for this phenomenon. In case of an oral transmission it might be that the lymphoreticular system in the gut acts as a barrier for type 1 conformational variants, in the sense that this tissue facilitates the replication of type 2 conformers. This scenario is depicted in Figure 3.

### Type Selection by the Host Genome

The polymorphisms in the prion protein gene of sheep provide a good example for a type selection in one species on the basis of the prion protein gene. Carrying the A_135_R_154_R_171_ allele seems to provide a certain resistance against classic scrapie for the respective animals in an affected flock. Therefore, selection towards this allele became the goal of breeding scrapie-resistant flocks (Fast and Groschup, 2013). However, when the apparently sporadically occurring atypical/Nor98 scrapie was more closely examined in the years following its discovery, it turned out that even sheep with two A_135_R_154_R_171_ alleles became sick (Buschmann et al., 2004; Lühken et al., 2007). In contrast, no sheep carrying the V_136_R_154_Q_171_/V_136_R_154_Q_171_ genotype were affected by atypical/Nor98 scrapie although this genotype had been considered the one most susceptible to classic scrapie. The A_135_R_154_Q_171_ allele conveys susceptibility to both scrapie forms, but the exchange of leucine for phenylalanine at codon 141 enhances susceptibility to atypical/Nor98 scrapie (Moum et al., 2005). So while the origin of disease for the individual sheep may differ for classic scrapie (oral and possibly *in utero* transmission Garza et al., 2011; van Keulen et al., 2008) and atypical/Nor98 scrapie (presumably sporadic origin Benestad et al., 2008) and this most certainly has an impact on selection as stated above, in this species the genotype plays a major role for susceptibility. Experimental transmission studies have given proof of this (Houston et al., 2002; Simmons et al., 2011, 2015). Interestingly, in sporadic CJD there is also a clear association between prion type and polymorphism: in type 1 patients, methionine at codon 129 is overrepresented while in type 2 patients this is the case with valine (Parchi et al., 1999). The earlier finding made by Goldfarb et al. (1992) that the methionine/valine polymorphism at Codon 129 decides if a patient with the D178N mutation will have either FFI or genetic CJD suggests a very strong selection of the host genome for a specific conformation. Here, the results are two unique disease phenotypes that are even addressed as different diseases (Goldfarb et al., 1992).

Host genetic factors may lead not only to a selection of conformers that propagate better than others, but can also provide a propagation stop, if there is no conformational variant in the cloud that is supported by the host. A species barrier is absolute, for example, or a polymorphism of the *PRNP* is selected that conveys resistance against the infection, as is the case with kuru. This acquired prion disease was widely spread among the Fore and neighboring groups of the Papua New Guinea highlands when described and examined in the 1950s (Gajdusek and Zigas, 1957). Mead et al. (2009) reported a novel *PRNP* polymorphism that “*was found exclusively in people who lived in the region in which kuru was prevalent and that was present in half of the otherwise susceptible women from the region of highest exposure who were homozygous for methionine at PRNP codon 129”*. They identified the G127V polymorphism as a prion disease resistance factor selected during the kuru epidemic. Moreover, the amino acid exchange in the prion protein seems to provide a complete resistance against the conversion of physiological prion protein by prion diseases in transgenic mouse models and “*acts as a potent dose-dependent inhibitor of wild-type prion propagation*” (Asante et al., 2015).

### How Many Strains Belong to One Type?

Even though conformational motifs are retained if propagation takes place in the new host, genetics of the host, including *PRNP* polymorphisms and other factors such as host-specific small RNAs and lipids, may produce more than one strain for one clinical phenotype, which is determined by the type. This is consistent with the fact that classic sheep scrapie (type 2), has been able to produce many different strains in the same inbred mouse lines (Bruce, 1993). Genetically different new hosts will produce different strains, of course. So one disease type will result in at least one strain, like BSE, per transmission to a host or result in many, like classic scrapie. BSE is really quite unique, as it produces the same two prion strains in RIII and VM mice, regardless of the particular cow source. In this it more resembles a laboratory prion strain, like e.g., 263K scrapie in hamsters. How many strains could be derived from sporadic CJD is difficult to determine, as an animal model does not exist that is susceptible for all human sporadic CJD phenotypes (Bishop et al., 2010; Head and Ironside, 2012).

### Summary: What Defines a Prion Type

A prion type is characterized by one or more structural motifs that determine key features of the clinical disease and neuropathological lesion profile in the original host (see Figures 1C, 2). Such are the deposition patterns of prion aggregates (synaptic or complex), the aggregate size and their stability towards denaturation that occur, regardless of the PRNP polymorphisms of the individual. Different conformational motifs also lead to different enzyme cleavage sites e.g., pK, and result in different western blot profiles. They are retained upon transmission to a new host, where they might result in several strains upon passaging, depending on further smaller changes of conformation in the original isolate mixture within the type and factors determined by the host (see above).

## Conclusion

The history of prion research is a long one, though the name *prion* is still comparatively young. Many experiments on laboratory animals and the stability of the infectious agent were undertaken before its proteinaceous nature was accepted by most scientists. Notably, while still working with the slow virus concept in the 1970s, Gajdusek (1977) already claimed a number of other neurodegenerative diseases such as AD, PD and Huntington’s disease and the spongiform encephalopathies (Kuru, CJD, scrapie and TME) to share pathogen-related properties due to the experiments he and others had performed. This provided an early foundation for today’s perception of neurodegenerative diseases. In the last 30–40 years terms such as strains and types have been used by researchers to describe the differences they found in naturally occurring and laboratory-derived diseases for many years, but the terminology has not been very accurate, especially regarding the application of “strains”. In the light of parallels drawn nowadays between prion diseases and other neurodegenerative diseases, an accurate terminology is even more important. The developments in prion research show us that the conformational characteristics found in the original host, which determine key features of the disease phenotype including the neuropathologic and molecular profile, characterize a protein aggregate type. Any modifications of the misfolding motifs that arise after transmission to and passaging in a new species due to variations in protein genome and host-specific molecules (such as host RNA and lipids) as well as a possible host-preference of certain conformers, will characterize protein aggregate strains (i.e., lesion profile, incubation period in prions and detailed immunohistochemical profile). One protein aggregate type may produce several strains after passaging in a new species. Serial passaging leads to stable strain properties. The route of transmission, i.e., intracerebral vs. oral, may contribute to a selection of aggregation motifs by the host and result in differences of efficacy in transmission. In principle, the results from prion research have already been replicated in animal experiments by using beta-amyloid and tau aggregates and an acceleration of seeding over time can be observed.

Overall, a lot can be learned from prion diseases as a model for protein aggregate-related neurodegenerative diseases and a better grasp of how types and strains evolve will help to unravel cause or consequence of protein aggregates in the pathophysiological processes of neurodegenerative diseases.

## Author Contributions

All three authors developed the concept of this review together, including the figures. WMW and WJS-S drafted the manuscript. All authors revised it critically and approved the final version.

## Conflict of Interest Statement

The authors declare that the research was conducted in the absence of any commercial or financial relationships that could be construed as a potential conflict of interest.

## Footnotes

^1^http://www.ictvonline.org/virusTaxInfo.asp
